# Monotherapy or combination antibiotic therapy in the treatment of Pseudomonas aeruginosa ventilator-associated pneumonia

**DOI:** 10.1186/s13054-023-04656-7

**Published:** 2023-09-22

**Authors:** Yanfei Shen, Xinfang Xie

**Affiliations:** 1 Department of Intensive Care, Zhejiang Hospital, 1229 Gudun Road, Hangzhou, 310013 Zhejiang China; 2 Department of Intensive Care, Yuhuan Second People’s Hospital of Health Community Group, 77 Huanbaozhong Road, Taizhou, Zhejiang China

To the Editor,

We read with great interest the recent study by Dr. Foucrier et al. [1], in which they compared the efficacy of combination antibiotic therapy versus monotherapy in ICU patients with Pseudomonas aeruginosa ventilator-associated pneumonia (PA-VAP). A total of 169 patients were included, and they reported that the patients in the combination therapy group had similar outcomes to those in the monotherapy group (20/75 (26.6%) vs. 17/94 (18.1%), *p* = 0.180). Antibiotic management plays a key role in critical care [2]. We would like to add some comments.

First, this study is a secondary analysis of a previous randomized controlled trial, in which the sample size was calculated based on the comparison between long- and short-term antibiotic therapy in PA-VAP patients. Therefore, it remains unclear whether the sample size in the current cohort study is sufficient to detect the difference in the impact of combination therapy versus monotherapy on mortality in PA-VAP patients. In the current study, although the crude comparison was non-significant, there was an increasing trend of mortality in combination therapy (20/75 (26.6%) vs. 17/94 (18.1%), *p* = 0.180). We cannot rule out the risk that this non-significant finding was affected by underpowered sample size.

Second, several studies have also investigated the efficacy of combination and monotherapy in PA-VAP. For instance, in a previous multicenter cohort study [3] including 183 PA-VAP patients, Dr. Montero et al. also reported that the overall mortality was comparable between the monotherapy and combination therapy groups, supporting the current study's findings. However, different from the present study, Dr. Montero et al. used the initial antibiotic therapy (when the pulmonary infection was suspected) as the grouping method (monotherapy or combination therapy), and all inappropriate therapies were adjusted according to the bacteria identification or antimicrobial susceptibility testing result. However, the current study's grouping method (definitive monotherapy or combination therapy) was based on the antibiotic regimens after adapting to the antimicrobial susceptibility testing result. Therefore, it is reasonable to infer that the difference in mortality between the monotherapy and combination therapy in the current study should be different from previous studies.

Third, in the current study, the definitive antibiotic therapy was left to the discretion of the physician according to usual care. In clinical practice [4], clinicians may be more likely to prescribe combination therapy for pandrug-resistant PA (PDR-PA) or difficult-to-treat resistance PA (DTR-PA) infection, which was also associated with increased mortality [5]. Therefore, in the multivariable analysis, we suggest that the proportion of patients with PDR-PA or DTR-PA also should be considered.

## Data Availability

Not applicable.
